# Supplementary material for: Cortical signatures of auditory looming bias show cue-specific adaptation between newborns and young adults
Source: Commun Psychol. 2024 Jun 8;2:56. doi: 10.1038/s44271-024-00105-5 (PMC11163589; doi:10.1038/s44271-024-00105-5)
Supplement: Supplementary file 2 — Reporting summary [file 44271_2024_105_MOESM2_ESM.pdf]

nature portfolio

Corresponding author(s): Karolina Ignatiadis and Robert Baumgartner

Last updated by author(s): May 22, 2024

Reporting Summary

Nature Portfolio wishes to improve the reproducibility of the work that we publish. This form provides structure for consistency and transparency in reporting. For further information on Nature Portfolio policies, see our Editorial Policies and the Editorial Policy Checklist.

Statistics

For all statistical analyses, confirm that the following items are present in the figure legend, table legend, main text, or Methods section.

n/a	Confirmed	
☐	☒	The exact sample size (*n*) for each experimental group/condition, given as a discrete number and unit of measurement
☐	☒	A statement on whether measurements were taken from distinct samples or whether the same sample was measured repeatedly
☐	☒	The statistical test(s) used AND whether they are one- or two-sided<br>*Only common tests should be described solely by name; describe more complex techniques in the Methods section.*
☐	☒	A description of all covariates tested
☐	☒	A description of any assumptions or corrections, such as tests of normality and adjustment for multiple comparisons
☐	☒	A full description of the statistical parameters including central tendency (e.g. means) or other basic estimates (e.g. regression coefficient) AND variation (e.g. standard deviation) or associated estimates of uncertainty (e.g. confidence intervals)
☐	☒	For null hypothesis testing, the test statistic (e.g. F, t, r) with confidence intervals, effect sizes, degrees of freedom and P value noted<br>*Give P values as exact values whenever suitable.*
☐	☒	For Bayesian analysis, information on the choice of priors and Markov chain Monte Carlo settings
☐	☒	For hierarchical and complex designs, identification of the appropriate level for tests and full reporting of outcomes
☐	☒	Estimates of effect sizes (e.g. Cohen's *d*, Pearson's *r*), indicating how they were calculated

Our web collection on statistics for biologists contains articles on many of the points above.

Software and code

Policy information about availability of computer code

Data collection	Code provided under https://osf.io/4gdy2/.
Data analysis	Code provided under https://osf.io/4gdy2/.

For manuscripts utilizing custom algorithms or software that are central to the research but not yet described in published literature, software must be made available to editors and reviewers. We strongly encourage code deposition in a community repository (e.g. GitHub). See the Nature Portfolio guidelines for submitting code & software for further information.

Data

Policy information about availability of data

All manuscripts must include a data availability statement. This statement should provide the following information, where applicable:

- Accession codes, unique identifiers, or web links for publicly available datasets
- A description of any restrictions on data availability
- For clinical datasets or third party data, please ensure that the statement adheres to our policy

Data are available under https://osf.io/4gdy2/.

Human research participants

Policy information about studies involving human research participants and Sex and Gender in Research.

Reporting on sex and gender	61 participants of the female sex (16 adult, 45 newborn) and 73 participants of the male sex (14 adult, 59 newborn) were considered in our study. Findings apply to both sexes. The factor of sex was considered in order to obtain a balanced design. Information was self-reported for the adult participants, and reported by the parents for the newborns. Information on gender was not collected. Sex- or gender-specific analyses were not performed as the factor was not implicated in our research questions.
Population characteristics	Healthy young adults with no self-reported indications of psychological and neurological disorders or acute or chronic heavy respiratory diseases that may prevent the participant from sitting still during the EEG recording. We initially measured participants' hearing thresholds between 1 and 12.5 kHz to ensure that they deviated not more than 20 dB from their age mean. Twenty-nine participants fulfilled this requirement and took part in the study (15 female sex: 25.0 ± 2.60 years old; 14 male sex: 25.1 ± 2.77 years old; reported as mean ± standard deviation). We recruited 104 healthy, full-term newborns (0–4 days of age, 59 male sex, 45 female sex). 46 were firstborns, 34 were second, 14 were third child and 6 had more than 3 siblings. None of them were twins. Mean gestational age was 40.17 ± 1 weeks and mean birth-weight 3787 ± 373 g. All newborns had normal hearing as indicated by successfully completing a Brainstem Evoked Response Audiometry (BERA) test prior to the experiment. No data on race / ethnicity was collected.
Recruitment	Adult participants were recruited through the institute's database of voluntary study participants. Recruitment of newborn participants took place at the maternity ward of the hospital. Pregnant women were contacted by a research nurse employed by RCNS.
Ethics oversight	This study followed Good Clinical Practice (GCP) standards and was conducted in full compliance with the Declaration of Helsinki (2000) and all applicable national and international laws. Adult testing was performed in accordance with the Austrian Universities Act of 2002 and the newborn protocol was also approved by the National Public Health Center, Hungary. Informed consent was obtained from adult participants and from either one or both parents of the newborn participants.

Note that full information on the approval of the study protocol must also be provided in the manuscript.

Field-specific reporting

Please select the one below that is the best fit for your research. If you are not sure, read the appropriate sections before making your selection.

☐ Life sciences ☒ Behavioural & social sciences ☐ Ecological, evolutionary & environmental sciences

For a reference copy of the document with all sections, see nature.com/documents/nr-reporting-summary-flat.pdf

Behavioural & social sciences study design

All studies must disclose on these points even when the disclosure is negative.

Study description	Quantitative study including two age groups (adults and newborns tested cross-sectionally) with two within subject factors manipulated either in passive or active conditions (only passive presentation was feasible for newborns).
Research sample	For young adult group, twenty-nine participants were included (15 female sex: 25.0 ± 2.60 years old; 14 male sex: 25.1 ± 2.77 years old; denoted as mean ± standard deviation). We aimed for a sample balanced over sex/gender. The young adults are representative for Austrian university students. For the newborn group, we recruited 104 healthy, full-term neonates (0–4 days of age, 59 male, 45 female). The sample is representative of the full-term population in terms of gestational age and birth weight. The hospital has a large catchment area (ca. 2600 births/year); the patient pool is representative of the population in Budapest. We aimed for a sample balanced over sex and representative of socioeconomic status.
Sampling strategy	To determine the appropriate sample size for our study, an a priori power analysis was conducted for a 2-sided mixed effect design at a 0.05 alpha level and desired power level of 0.80 with medium effect sizes (0.40). This resulted in an estimated sample size of N= 28 for each of the two age groups. To compensate for potential exclusions, 30 adults and 90 newborns were finally recruited.
Data collection	Recruitment of newborns was carried out at the maternity ward of the hospital by a research nurse employed by RCNS.
Timing	Data collection of the adult data was performed continuously in spring 2021. Newborn data was collected continuously over 2021-2022 (occasionally interrupted due to COVID restrictions or due to other studies).
Data exclusions	The exclusion criteria for all studies included a history of psychiatric or neurological symptoms; the usage of recreational drug or medication with any psychoactive effects; and the existence of non-removable metallic parts that would prevent MRI scanning. Adult participants were screened to have pure-tone thresholds within normal age-dependent limits. All newborns had normal hearing as indicated by successfully completing a Brainstem Evoked Response Audiometry (BERA) test. Exclusion of newborn data was based on

	the proportion of useful trials: after data preprocessing, 33 subjects maintained less than 60 % of the trials and were therefore excluded; 71 newborn data sets were finally analyzed for the present study.
Non-participation	Not recorded
Randomization	Participants were allocated into two groups based on their age (newborn, young adult).

Reporting for specific materials, systems and methods

We require information from authors about some types of materials, experimental systems and methods used in many studies. Here, indicate whether each material, system or method listed is relevant to your study. If you are not sure if a list item applies to your research, read the appropriate section before selecting a response.

Materials & experimental systems

n/a	Involved in the study
☒	☐ Antibodies
☒	☐ Eukaryotic cell lines
☒	☐ Palaeontology and archaeology
☒	☐ Animals and other organisms
☒	☐ Clinical data
☒	☐ Dual use research of concern

Methods

n/a	Involved in the study
☒	☐ ChIP-seq
☒	☐ Flow cytometry
☐	☒ MRI-based neuroimaging

Magnetic resonance imaging

Experimental design

Design type	Resting state
Design specifications	Structural anatomical data was recorded only for EEG source localization purposes.
Behavioral performance measures	none

Acquisition

Imaging type(s)	Structural
Field strength	3 Tesla
Sequence & imaging parameters	not relevant
Area of acquisition	not relevant

Diffusion MRI ☐ Used ☒ Not used

Preprocessing

Preprocessing software	MRIcroGL 1.2.20201102, Freesurfer reconstruction
Normalization	not relevant
Normalization template	not relevant
Noise and artifact removal	not relevant
Volume censoring	not relavant

Statistical modeling & inference

Model type and settings	none
Effect(s) tested	none

Specify type of analysis: ☐ Whole brain ☒ ROI-based ☐ Both

Anatomical location(s): Desikan-Killiany atlas as implemented in Brainstorm.

Statistic type for inference (See Eklund et al. 2016)	none
Correction	none

Models & analysis

n/a	Involved in the study
☒	☐ Functional and/or effective connectivity
☒	☐ Graph analysis
☒	☐ Multivariate modeling or predictive analysis
